# Plasma Clusterin (ApoJ) Levels Are Associated with Adiposity and Systemic Inflammation

**DOI:** 10.1371/journal.pone.0103351

**Published:** 2014-07-30

**Authors:** Jong Chul Won, Cheol-Young Park, Sang Woo Oh, Eon Sook Lee, Byung-Soo Youn, Min-Seon Kim

**Affiliations:** 1 Department of Internal Medicine, Cardiovascular and Metabolic Disease Center, Sanggye Paik Hospital, Inje University College of Medicine, Seoul, Korea; 2 Department of Internal Medicine, Kangbuk Samsung Hospital, Sungkyunkwan University School of Medicine, Seoul, Korea; 3 Department of Family Medicine, Dongguk University Ilsan Hospital, Ilsan, Korea; 4 Department of Family Medicine, Center for Health Promotion, Ilsan Paik Hospital, Inje University College of Medicine, Ilsan, Korea; 5 Department of Anatomy, School of Medicine, Wonkang University, Iksan, Jeonbuk, Korea; 6 Department of Internal Medicine, Asan Medical Center University of Ulsan College of Medicine, Seoul, Korea; The University of Hong Kong, Hong Kong

## Abstract

Obesity and insulin resistance are hallmarks of the metabolic syndrome, which is associated with low-grade chronic inflammation. Clusterin/apolipoprotein J is an abundant plasma chaperone protein that has recently been suggested as a potential biomarker that reflects the inflammatory process in Alzheimer's disease. In the present study, we investigated anthropometric and clinical factors affecting the plasma levels of clusterin in healthy Korean subjects. We measured fasting plasma clusterin levels in healthy Korean adults (111 men and 93 women) using ELISA kit. We analyzed the relationship between plasma clusterin concentrations and anthropometric and clinical parameters. Fasting plasma clusterin concentrations were higher in overweight and obese subjects than in lean subjects. Correlation analysis revealed that the plasma clusterin levels were positively associated with indices of obesity such as body mass index (BMI), waist circumference and waist-hip ratio and markers of systemic inflammation such as high sensitivity C-reactive protein (hsCRP), uric acid, ferritin and retinol binding protein-4. Multiple linear regression analysis showed that sex, BMI and hsCRP were independent determinants of plasma clusterin levels. Furthermore, plasma clusterin levels showed an upward trend with increasing numbers of metabolic syndrome components. These findings suggest that fasting plasma clusterin levels correlate with the parameters of adiposity and systemic inflammation in healthy adults. Therefore, the circulating clusterin level may be a surrogate marker for obesity-associated systemic inflammation.

## Introduction

Cumulative data have demonstrated that obesity is accompanied by low-grade inflammatory responses in the circulation and within metabolic organs [1]. Excessive fat deposition contributes to systemic inflammation by releasing free fatty acids, proinflammatory adipokines such as tumor necrosis factor (TNF)-α, plasminogen activator inhibitor-1, resistin, and retinol binding protein (RBP)-4 [2]. Chronic inflammation has also been shown to bridge obesity and the development of insulin resistance, metabolic syndrome, and type 2 diabetes mellitus (T2DM) [1], [3]. These diseases play a fundamental role in the initiation and progression of atherosclerosis and subsequent cardiovascular disease [4].

Clusterin (also named apolipoprotein J, sulfated glycoprotein-2, androgen repressed protein, and complement lysis inhibitor) is a heterodimeric glycoprotein in which α and β chains are interconnected via 5 disulfide bonds [5]. Clusterin is ubiquitously expressed in various tissues including the liver, brain, ovary, testis, heart, and blood vessels [6]. The secretory form of clusterin is also abundant in biological fluids such as plasma, seminal fluid, milk and cerebrospinal fluid [6]. In the circulation, clusterin is known as a component of the lipid-poor subclass of high density lipoprotein-cholesterol (HDL-C), which contains apoA-1 and paraoxonase [7].

Clusterin has been implicated in a number of biological processes such as immune modulation, sperm maturation, lipid transportation, and cancer cell survival [6], [8]. In particular, clusterin plays a significant role in inflammation and immune responses through molecular interactions with complement factors, immunoglobulins, transforming growth factor (TGF)-β, phosphorylated IκBα, and activated Bax [9]. Large scaled genome-wide association studies in Caucasian and Asian populations have demonstrated a strong association between the single nucleotide polymorphisms (SNPs) in the clusterin gene and Alzheimer's disease [10], [11], [12]. Moreover, a recent study has shown that increased plasma clusterin concentrations are significantly related to the severity and progression of Alzheimer's disease [13], suggesting that plasma clusterin as a potential peripheral biomarker of Alzheimer's disease. Since Alzheimer's disease is more prevalent in subjects with middle-aged obesity and insulin resistance [14], [15], we investigated the relationship between the plasma clusterin levels and the parameters of adiposity, metabolic syndrome, and systemic inflammation in healthy Korean subjects.

## Methods

### Subjects

Among Korean adults, who visited Paik Hospital (Ilsan, Korea) for a health screen in 2005 and voluntarily participated to this study, 111 males and 93 females were enrolled in data analysis as they met the following inclusion criteria. The subject was 19∼70 years old and healthy as judged by a responsible physician on the bases of medical evaluation including medical history and physical examination, non-pregnant in female, and did not take any medication at time of the study. All subjects fulfilled the following exclusion criteria: 1) absence of acute inflammatory disease or infection, 2) no clinical evidence of other medical conditions, such as hypertension, diabetes mellitus, pulmonary, cardiovascular, liver and renal diseases, alcohol or drug abuse and pregnancy. The Institutional Review Boards at the Ilsan Paik Hospital approved the study protocol according to the Declaration of Helsinki. All participants signed written informed consent. They completed a self-administered questionnaire that included demographic characteristics, general health status, alcohol and smoking history, and current medications.

### Measurements of clinical, laboratory, and anthropometric parameters

Measurements of anthropometric parameters were performed for the study subjects before breakfast when wearing light clothing and no shoes. The heights and weights were measured. The body mass index (BMI) was calculated as the weight/height^2^ (kg/m^2^). Lean, overweight or obesity was defined by a BMI of 18.5∼22.9, 23.0∼24.9, or ≥25, respectively, in accordance with the definitions for Asian adults proposed by the WHO Regional Office for the Western Pacific (WPRO) [16]. Waist circumference (WC) was measured midpoint between the lowest rib and the iliac crest. Hip circumference (HC) was measured around the pelvis at the point of maximal protrusion of the buttocks. The waist and hip circumferences were measured to the nearest 0.1 cm. Blood pressure was measured from the right arm after a 20 minute-rest. Mean values of two consecutive blood pressure recordings were used for statistical analysis. The subjects who smoked regularly during the previous 12 months were classified as current smokers.

Blood samples were collected from all subjects after an overnight 10 hour fast (between 0830 and 1030). Plasma was immediately separated and stored at −70°C until analysis. The fasting plasma glucose, total cholesterol (TC), triglyceride (TG), HDL-C and uric acid levels were measured via enzymatic methods using an auto-analyzer (Hitachi, Tokyo, Japan). Plasma high-sensitivity C-reactive protein (hsCRP) levels were measured using an automated particle-enhanced immunoturbidimetric assay (Roche Diagnostics, Indianapolis, IN). Plasma ferritin levels were measured by electrochemiluminescence immunoassay (Roche Diagnostics). Plasma insulin concentrations were assayed using radioimmunoassay (RIA, Roche Diagnostics). Insulin resistance was determined using the homeostasis model assessment of insulin resistance (HOMA-IR) [17]. Plasma concentrations of leptin were determined using a RIA kit (Linco Research, St. Charles, MO). Plasma concentrations of adiponectin and RBP4 were also measured by RIA (AdipoGen, Incheon, Korea).

Clusterin concentrations in the plasma were assayed using ELISA (AdipoGen), in which a polyclonal antibody against human full-length secretary clusterin was used. The assay was performed according to a commercial protocol (www.adipogen.com). Intra- and inter-assay variations were 5∼10% and 7∼10%, respectively. To avoid inter-assay variation, we assayed all of the samples at one time. The mean values of duplicate measurements were used for statistical analysis.

### Diagnosis of metabolic syndrome

We diagnosed metabolic syndrome by the criteria of the National Cholesterol Education Program (NCEP) Adult Treatment Panel III [18]. Central obesity was defined as that waist circumference was ≥90 cm for Asian men and ≥80 cm for Asian women proposed by WPRO [19].

### Statistical analyses

Variables were presented as mean ± SD or mean ± SE. Variables showing a skewed distribution were expressed as median with range or log-transformed before statistical analysis. Student's *t* test or χ^2^ test, or ANOVA were performed to compare variables between groups as appropriate. Age- and/or sex-adjusted comparisons were performed using analysis of covariance (ANCOVA). To evaluate the association between plasma clusterin levels and other variables, we performed Pearson's correlation coefficients analyses and then multiple linear regression analyses using the stepwise variable selection method. Multicollinearity was assessed using the variance inflation factor (VIF). To avoid multicollinearity between WC and waist-hip ratio (WHR), WHR included as independent variable. In this model, no evidence for multicollinearity was observed. All VIFs were below 1.5. The test for trend was performed using polynomial contrast procedure. All significance tests were two-sided, and the results were considered as statistically significant at *P* value <0.05. Data were analyzed using SPSS for Windows (Version 12.0, SPSS Inc., Chicago, IL).

## Results

### Characteristics of the study subjects

The clinical characteristics of the study subjects are listed in Table 1. The average BMI was 25.1 kg/m^2^ (range, 15.5∼33.5 kg/m^2^) and average WC was 87 cm (range, 62∼112 cm). Thirty eight subjects (17 male, 21 female) were lean, whereas 166 subjects (94 male, 72 female) were classified as overweight or obese. None of subjects was using lipid-lowering therapy or had diabetes.

**Table 1:** Clinical, anthropometric and biochemical characteristics of the study subjects.

Subjects	All	Lean	Overweight/obese
	(*n* = 204)	(*n* = 38)	(*n* = 166)
Sex (M/F)	111/93	17/21	94/72
Age (year)	40±10	37±11	40±10
Metabolic syndrome (n, %)	53 (26.0)	2 (5.2)	51 (30.7)
Smoker (n, %)	58 (28.4)	10 (27.8)	48 (29.5)
BMI (kg/m^2^)	25.1±2.9	21.2±1.5	26.0±2.3*
WC (cm)	86.6±8.2	78.1±6.2	88.6±7.3*
WHR	0.86±0.06	0.82±0.06	0.87±0.05*
SBP (mmHg)	117±14	108±11	119±14*
DBP (mmHg)	72±94	65±7.8	73±9.2*
FPG (mmol/L)	5.0±0.5	5.0±0.5	5.0±0.5
FPI (pmol/L)§	44.5±31.1	33.8±31.6	47.0±30.5*
HOMA-IR§	1.4±1.0	1.1±1.0	1.5±1.0*
TC (mmol/L)	4.7±0.8	4.6±0.7	4.8±0.8
TG (mmol/L)§	1.9±1.3	1.5±1.0	2.0±1.4*
HDL-C (mmol/L)	1.2±0.3	1.4±0.4	1.1±0.3*
hsCRP (mg/L)‡ ^,^ §	0.47 (5.66)	0.26 (3.69)	0.52 (5.65)*
Uric acid (µmol/L)‡	336 (414)	288 (384)	354 (414)†
Ferritin (µg/L)‡	78.8 (15.2∼396.6)	50.2 (15.2∼376.2)	88 (18.4∼396.6)†
Leptin (ng/mL)§	6.7±6.6	5.0±4.0	7.6±6.3*
Adiponectin (µg/mL)§	7.0±5.0	7.4±3.5	6.9±5.3†
RBP4 (µg/mL)§	60.6±26.2	54.5±26.8	62.0±26.0†

Data are the mean ± SD.

‡ Data are median values and range.

§ Logarithmic transformation was performed before the analysis.

*P<0.01,

† *P*<0.05 *vs.* lean. BMI, body mass index; WC, waist circumference; WHR, waist-hip ratio; SBP, systolic blood pressure; DBP, diastolic blood pressure;: FPG, fasting plasma glucose; FPI, fasting plasma insulin; HOMA-IR homeostasis model of insulin resistance; TC, fasting plasma total cholesterol; TG, fasting plasma triglyceride; HDL-C, high density lipoprotein cholesterol; hsCRP, high-sensitivity C-reactive protein; RBP4, retinol binding protein-4.

The overweight/obese subjects had a higher WC, WHR, and systolic and diastolic blood pressure, than the lean subjects. Fasting blood levels of insulin, TG, hsCRP, uric acid, ferritin, leptin and RBP4 were higher whereas fasting blood levels of HDL-C and adiponectin were lower in overweight/obese subjects compared with lean subjects. There was no difference in the percentage of smoker and fasting blood glucose levels between the two groups (Table 1).

### Plasma clusterin levels are higher in men and in overweight/obese subjects

The mean fasting plasma clusterin concentration in all subjects was 50.8±12.0 µg/mL (range, 22.6∼87.2 µg/mL). Plasma clusterin levels were significantly higher in men than in women (54±10 *vs*. 47±13 µg/mL, *P*<0.05). Overweigh/obese subjects had higher circulating clusterin levels compared with lean subjects (52.2±11.9 *vs.* 44.4±10.5 µg/mL, *P*<0.001). Age-adjusted plasma clusterin levels were significantly increased in overweigh/obese women compared to lean women (Table 2). In men, they tended to be higher in overweigh subjects and were significantly higher in obese subjects than in lean subjects (Table 2). Smokers had increased plasma clusterin levels compared to non-smokers (55.1±10.9 *vs.* 49.0±12.0 µg/mL, *P*<0.001).

**Table 2:** Age-adjusted fasting plasma clusterin concentrations in lean, overweight, and obese subjects.

µg/mL	Lean	Overweight	Obese	*P*-value
Total (*n*)	44.2±1.9 (38)	49.7±1.4* (65)	54.0±1.2* ^,^ † (101)	< 0.001
Men (*n*)	49.9±2.4 (17)	51.3±1.8 (32)	56.7±1.3* ^,^ † (62)	0.010
Women (*n*)	39.3±2.8 (21)	48.4±2.2* (33)	49.5±2.0* (39)	0.012

Data are mean ± SE.

*P<0.05 *vs*. lean,

† *P*<0.05 *vs*. overweight.

### Correlation between circulating clusterin levels and anthropometric and clinical parameters

Pearson's correlation analysis showed that plasma clusterin levels positively correlated with BMI, WC, WHR, TC/HDL-C, TG/HDL-C, hsCRP, uric acid, ferritin, and RBP4 (Table 3). As plasma clusterin levels were affected by sex, correlation analysis was separately performed in men and women. In men, plasma clusterin levels showed a positive association with hsCRP and BMI (Table 3). In women, they significantly correlated with BMI and plasma leptin levels and marginally correlated with hsCRP (Table 3). Stepwise multiple linear regression analysis revealed that sex, hsCRP and BMI were the independent contributors affecting fasting plasma clusterin levels (Table 4).

**Table 3:** Correlation between fasting plasma clusterin concentrations and anthropometric and clinical parameters.

Variables	All		Men		Women	
	γ	*P*	γ	*P*	γ	*P*
Age	−0.05	0.520	0.04	0.712	−0.06	0.579
BMI	0.22	0.002	0.21	0.031	0.21	0.040
WC	0.25	<0.001	0.14	0.152	0.13	0.213
WHR	0.20	0.004	0.16	0.094	−0.01	0.973
SBP	0.05	0.468	0.16	0.589	−0.16	0.122
DBP	0.13	0.066	0.13	0.180	−0.09	0.391
FPG	0.02	0.826	−0.04	0.711	−0.03	0.794
FPI§	0.09	0.228	0.14	0.155	0.07	0.483
HOMA-IR§	0.07	0.322	0.13	0.189	0.07	0.518
TC/HDL-C	0.14	0.047	0.09	0.371	−0.01	0.987
TG/HDL-C§	0.17	0.016	0.08	0.403	0.09	0.423
hsCRP§	0.21	0.003	0.23	0.016	0.20	0.053
Uric acid	0.17	0.017	−0.07	0.474	−0.02	0.870
Ferritin	0.19	0.007	0.02	0.851	0.01	0.952
Leptin§	−0.10	0.197	0.17	0.094	0.21	0.049
Adiponectin§	−0.10	0.159	0.01	0.905	0.02	0.833
RBP4§	0.23	0.001	0.10	0.307	0.16	0.127

γ: Pearson's coefficient.

§ Logarithmic transformation was performed before analysis. BMI, body mass index; WC, waist circumference; WHR, waist-hip ratio; SBP, systolic blood pressure; DBP, diastolic blood pressure; FPG, fasting plasma glucose; FPI, fasting plasma insulin; HOMA-IR homeostasis model of insulin resistance; TC, fasting plasma total cholesterol; TG, fasting plasma triglyceride; HDL-C, high density lipoprotein cholesterol; hsCRP, high-sensitivity C-reactive protein; RBP4, retinol binding protein-4.

**Table 4:** Independent variables affecting plasma clusterin concentration in stepwise multivariate analysis.

	Unstandardized coefficients		Standardized coefficients	*P*-value
	*β*	SE	*β*	
Constant	65.91	24.87		
Women	−13.87	3.38	−0.59	<0.01
hsCRP§	2.51	0.99	0.20	0.01
BMI	0.80	0.39	0.21	0.03

*R* ^2^(coefficient of determination) for the model: 0.245. Multiple linear regression analysis was performed in 204 subjects. Data for HOMA-IR, TG/HDL-C, hsCRP, leptin, adiponectin, and RBP4 were log-transformed due to skewed distributions. In stepwise regression analysis, the independent variables tested initially were age, sex, smoking status, BMI, WHR, DBP, FPG, FPI, HOMA-IR, TC/HDL-C, TG/HDL-C, hsCRP, uric acid, ferritin, leptin, adiponectin, and RBP4 (abbreviations in Table 1 and 3). No combination with a strong correlation (*R*>0.9) was found among these variables after excluding HOMA-IR. Multiple regression analysis was performed among the remaining independent variables.

§ Logarithmic transformation was performed before analysis.

### Analysis of clinical, anthropometric and metabolic parameters according to plasma clusterin levels

We subgrouped the subjects into 4 groups according to the quartiles of plasma clusterin levels and compared the clinical, anthropometric and metabolic characteristics between 4 groups. There were more men in the highest quartile for clusterin than in any of the other 3 groups (Table 5). The subjects in the highest quartile for age- and sex-adjusted clusterin levels had a higher BMI, fasting blood glucose, hsCRP and leptin concentrations compared with the subjects in the lowest clusterin quartile (Table 5).

**Table 5:** Comparison of sex- and age-adjusted clinical, anthropometric and laboratory characteristics according to the quartiles of plasma clusterin concentrations.

	1^st^ quartile (22.6∼41.8)	2^nd^ quartile (42.2∼51.3)	3^rd^ quartile (51.4∼57.3)	4^th^ quartile (57.6∼87.2)	*P*-value
Men (*n*, %)	13 (25.4)	28 (15.7)	33 (64.7)	37 (72.5)	<0.001
Age (years)	40.5±1.5	39.1±1.7	39.3±1.4	40.7±1.4	0.836
Metabolic syndrome (%)	15.4±0.6	25.9±0.6	27.8±0.6	34.9±0.6	0.157
Smoker (%)	25.4±0.6	29.5±0.6	24.3±0.6	37.9±0.6	0.362
BMI (kg/m^2^)	24.2±0.4	25.2±0.4	25.3±0.4	25.9±0.4	0.036
WC (cm)	84.5±1.0	87.0±1.0	87.7±1.0	87.2±1.0	0.127
WHR	0.86±0.01	0.86±0.01	0.86±0.01	0.87±0.01	0.806
SBP (mmHg)	118.9±2.0	116.6±1.9	115.3±1.9	117.7±2.0	0.601
DBP (mmHg)	72.5±1.2	71.9±1.2	70.9±1.2	73.1±1.2	0.567
FPG (mmol/L)	5.1±0.1	5.0±0.1	4.8±0.1	5.1±0.1	0.035
FPI (pmol/L)§	42.0±4.6	44.6±4.4	45.1±4.4	46.4±4.5	0.740
HOMA-IR§	1.4±0.2	1.4±0.1	1.4±0.2	1.5±0.2	0.727
TC/HDL-C	4.4±0.2	4.0±0.2	4.4±0.2	4.4±0.2	0.133
TG/HDL-C§	3.3±0.4	2.6±0.4	3.4±0.4	3.4±0.4	0.485
hsCRP (mg/L)§	0.65±0.14	0.91±0.14	0.85±0.14	1.08±0.14	0.030
Uric acid (µmol/L)§	336.7±10.0	346.9±9.5	342.8±9.6	335.1±9.7	0.844
Ferritin (µg/L)	94.2±8.6	83.8±8.3	92.4±8.3	98.2±8.5	0.662
Leptin (ng/mL)§	6.8±0.7	6.5±0.7	6.8±0.7	8.4±0.7	0.047
Adiponectin (µg/mL)§	7.1±0.8	6.4±0.6	7.6±0.7	6.9±0.7	0.599
RBP4 (µg/mL)§	57.7±3.6	58.5±3.5	62.6±3.5	63.8±3.6	0.224

Data are mean ± SE. Age- and sex-adjusted comparisons were performed using analysis of covariance (ANCOVA) which provides a statistical significance (*P* value) in differences between the groups after adjustment for the covariates (age and sex).

§ Logarithmic transformation was performed before analysis. BMI, body mass index; WC, waist circumference; WHR, waist-hip ratio; SBP, systolic blood pressure; DBP, diastolic blood pressure; FPG, fasting plasma glucose; FPI, fasting plasma insulin; HOMA-IR homeostasis model of insulin resistance; TC, fasting plasma total cholesterol; TG, fasting plasma triglyceride; HDL-C, high density lipoprotein cholesterol; hsCRP, high-sensitivity C-reactive protein; RBP4, retinol binding protein-4.

### Plasma clusterin levels and metabolic syndrome

**Figure 1:**
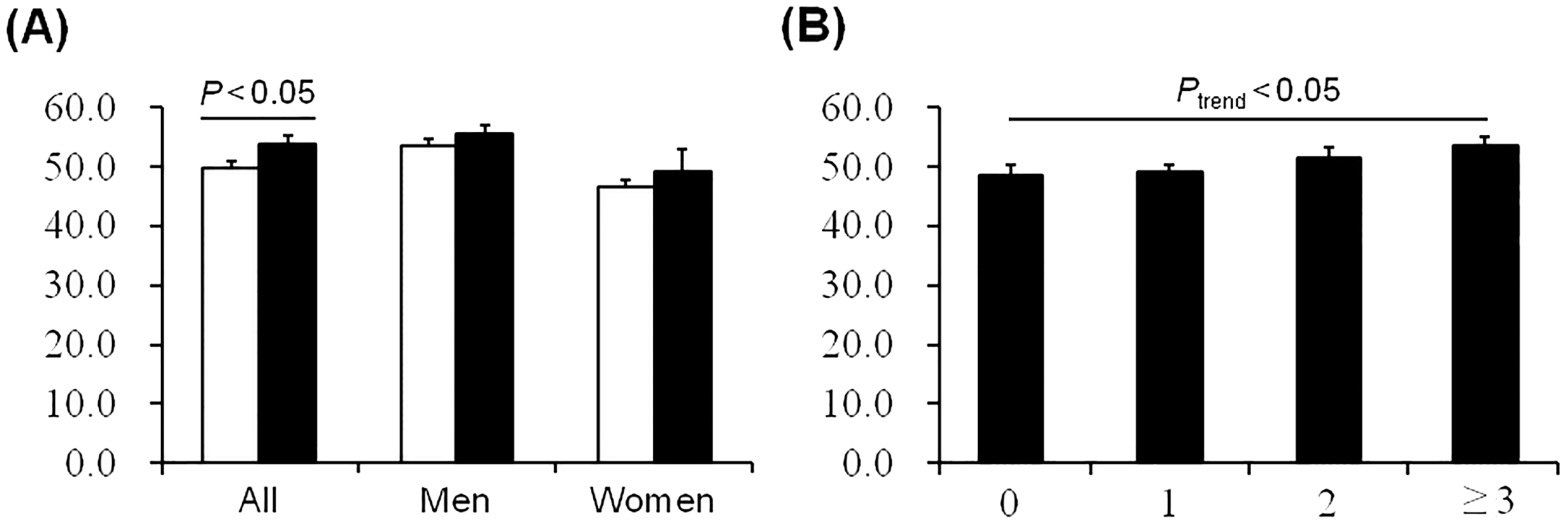
Relationship between plasma clusterin levels and metabolic syndrome. (A) Fasting plasma clusterin concentrations in subjects with and without metabolic syndrome. White bars: subjects without metabolic syndrome (*n* = 151 for all, 73 for men, and 78 for women), Black bars: subjects with metabolic syndrome (*n* = 53 for all, 38 for men, and 15 for women). (B) Plasma clusterin concentrations in relation to increases in the number of metabolic syndrome components. The breakdown of the number of components in the study cohort was as follows: 0 (*n* = 39), 1 (*n* = 57), 2 (*n* = 55) and 3 or more (*n* = 53). Data are the mean ± SE.

Plasma clusterin levels were higher in subjects with metabolic syndrome than in those without metabolic syndrome (Fig. 1A). Sex-stratified analysis also revealed a tendency of increase in circulating clusterin levels in individuals with metabolic syndrome (Fig. 1A). Furthermore, plasma clusterin levels showed an upward trend with increased metabolic syndrome component numbers (Fig. 1B).

## Discussion

In the present study, plasma clusterin levels were positively associated with systemic inflammation markers in apparently healthy subjects such as hsCRP [20], uric acid [21], ferritin [22], and RBP4 [23] and independently correlated with a key inflammation biomarker hsCRP in multiple regression analysis. Moreover, plasma clusterin concentrations were higher in the subjects with metabolic syndrome and increased with the number of metabolic syndrome parameters. Since metabolic syndrome accompany low-grade chronic inflammation [24], [25], our findings may suggest that plasma clusterin levels reflect systemic meta-inflammation. Recently, it has been shown that plasma clusterin correlates with the severity and progression of Alzheimer's disease [13]. Besides clusterin, various inflammatory proteins and cytokine levels are increased in the blood and cerebrospinal fluid of Alzheimer's disease patients [26]. Taken together, these results suggest that circulating clusterin levels might be related to the proinflammatory state associated with increasing adiposity, and strengthen that plasma clusterin is a potential biomarker of inflammation-related human disease.

In our current analyses, fasting plasma clusterin levels were found to be positively correlated with BMI, a parameter of whole body adiposity in healthy adults. The positive relationship between BMI and plasma clusterin levels was observed in both males and females. In contrast, previous studies have reported no significant correlation between BMI and plasma clusterin [27], [28]. This discrepancy may be due to differences in the cohorts studied, and in the clusterin assays and blood samples used. In an earlier study by Kujiraoka et al., the serum clusterin levels did not correlate with the BMI, but positively correlated with the paraoxonase levels in healthy subjects, and with the blood glucose levels in subjects with T2DM [27]. Since the subjects in the Kujiraoka's study cohort had a narrow BMI range (23.1±0.3 kg/m^2^), the effects of adiposity on clusterin levels might have been missed. Another previous study has reported that the plasma clusterin levels did not differ between obese and lean adolescents [28]. Interestingly, in that study, plasma clusterin levels were significantly reduced after weight reduction.

In addition to BMI, we found in our current analysis that the fasting clusterin levels were associated with markers of visceral adiposity, a principal metabolic syndrome component [29]. The subjects in our study cohort with metabolic syndrome showed higher circulating clusterin levels compared with those without metabolic syndrome. Sex-stratified analysis also displayed a tendency of increased clusterin levels in subjects with metabolic syndrome. Furthermore, higher number of metabolic syndrome components correlated with the higher plasma clusterin levels. Conversely, metabolic syndrome was found to be more common in subjects in the highest plasma clusterin quartile. These findings are consistent with the findings of the previous studies demonstrating increased circulating clusterin concentrations in patients with CVD and T2DM [27], [30], [31].

In our study, plasma clusterin levels were higher in men than in women. This finding was partly in agreement with the previous study showing that clusterin levels were slightly higher in men than in women [27]. Metabolic syndrome and smoking was more prevalent in men than in women, which may contribute to the increased circulating clusterin levels in men. However, sex was an independent variable affecting plasma clusterin levels in our cohort. Mechanisms underlying the sexual difference in plasma clusterin concentrations are yet to be identified.

Although the major source of circulating clusterin remains unclear, it has been well established that human hepatocytes secrete clusterin [32]. Clusterin is also expressed in various tissues such as brain, ovary, testis, heart, and blood vessels [6], from which it may be secreted to the circulation. Hepatic clusterin mRNA expression and serum clusterin levels have also been shown to be remarkably elevated after exposure to endotoxin (LPS), TNF-α and interleukin-1 in Syrian hamsters [33]. Hence, it can be reasonably speculated that obesity-associated systemic inflammation may induce the increased production or secretion of clusterin from the liver. On the other hand, obesity was reported to be independently associated with oxidative stress in a large-scale epidemiologic study [34]. Indeed, clusterin is an extremely sensitive biomarker of oxidative stress [8].

Plasma clusterin is associated with paraoxonase-1 as a component of HDL-C [27], [35]. In the atherosclerotic vascular lesions, clusterin is co-localized with paraoxonase and apolipoprotein A-1 [36]. Moreover, clusterin overexpression in injured blood vessels using adenovirus inhibits the migration, adhesion and proliferation of vascular smooth muscle cells and exerts protective effects on endothelial cells [37]. These data may suggest that clusterin has a protective role against vascular inflammation and atherosclerosis. In contrast, a deficiency of clusterin gene in rodents reduces neointimal hyperplasia [38]. Thus, whether increased clusterin levels have beneficial or adverse effects on the cardiovascular system remains inconclusive.

This study has some limitations. Because of cross-sectional design of this study, we couldn't determine the causality of the observed relationship between plasma clusterin levels and the anthropometric and clinical variables. Second, we could not estimate an appropriate sample size in this cross-sectional study. Indeed, an important limitation is the relatively small number of subjects. In addition, the subjects in this study were a narrow range of anthropometric and clinical indices, which may limit the power to represent the result of this study to general population. However, the purpose of this study was to investigate the association of circulating levels of clusterin and parameters reflecting the usual health status in non-diseased subjects. Therefore the findings of this study may provide the basis for understanding the role of clusterin in subjects with low-grade chronic inflammation such as in metabolic syndrome. Future studies will be needed to extend our observations to larger groups of subjects with a wide range of anthropometric and clinical indices, and with various inflammation-related diseases.

In summary, the circulating clusterin levels were significantly associated with the parameters for adiposity and systemic inflammation and increased in subjects with metabolic syndrome. Hence, plasma clusterin may be a potential biomarker of systemic meta-inflammation.
